# NF-κB transcription factor role in consolidation and reconsolidation of persistent memories

**DOI:** 10.3389/fnmol.2015.00050

**Published:** 2015-09-09

**Authors:** Verónica de la Fuente, Noel Federman, Gisela Zalcman, Angeles Salles, Ramiro Freudenthal, Arturo Romano

**Affiliations:** Laboratorio de Neurobiología de la Memoria, Departamento de Fisiología, Biología Molecular y Celular, Facultad de Ciencias Exactas y Naturales, Instituto de Fisiología, Biología Molecular y Neurociencias (IFIBYNE, UBA-CONICET), Universidad de Buenos Aires, Ciudad UniversitariaBuenos Aires, Argentina

**Keywords:** NF-κB, transcription factors, consolidation, reconsolidation, memory

## Abstract

Transcriptional regulation is an important molecular process required for long-term neural plasticity and long-term memory (LTM) formation. Thus, one main interest in molecular neuroscience in the last decades has been the identification of transcription factors that are involved in memory processes. Among them, the nuclear factor κB (NF-κB) family of transcription factors has gained interest due to a significant body of evidence that supports a key role of these proteins in synaptic plasticity and memory. In recent years, the interest was particularly reinforced because NF-κB was characterized as an important regulator of synaptogenesis. This function may be explained by its participation in synapse to nucleus communication, as well as a possible local role at the synapse. This review provides an overview of experimental work obtained in the last years, showing the essential role of this transcription factor in memory processes in different learning tasks in mammals. We focus the review on the consolidation and reconsolidation memory phases as well as on the regulation of immediate-early and late genes by epigenetic mechanisms that determine enduring forms of memories.

## Transcription Factors and Memory

Early in the research on the molecular basis of memory it was proposed that memory consolidation is a period during which new proteins must be synthesized (Davis and Squire, 1984; Goelet et al., 1986). The first evidence came from the use of antibiotics like puromycin and cycloheximide (Flexner et al., 1963; Agranoff and Klinger, 1964; Barondes and Cohen, 1966, 1967), which are protein synthesis inhibitors. These inhibitors were effective when administered shortly before or after acquisition, and induced memory impairment only when tested at long-term but not at short-term periods. Almost at the same time of these findings with protein synthesis inhibitors, the role of mRNA synthesis in memory formation was initially evidenced by the use of the antibiotic actinomycin D, which interferes with transcription (Barondes and Jarvik, 1964; Appel, 1965; Cohen and Barondes, 1966). Like the other antibiotics, actinomycin D impaired long-term memory (LTM) only when administered around training session, and the amnesic effect was manifested when tested at long-term periods. Many years later, the first transcription factors involved in synaptic plasticity, cAMP response element binding protein (CREB; Dash et al., 1990) and in memory consolidation, c-Fos (a member of the activated protein 1-AP-1-family; Anokhin and Rose, 1991) were identified. Subsequent work on CREB, from a series of studies in *Aplysia*, *Drosophila* and rodents, supported that this transcription factor takes part in an evolutionarily conserved molecular mechanism involved in neural plasticity associated with memory formation (Kaang et al., 1993; Bourtchuladze et al., 1994; Yin et al., 1994).

The identification of other transcription factors involved in neural plasticity and memory was achieved in the following years, such as CAAT enhancer binding protein (C/EBP; Alberini et al., 1994), zinc finger inducing factor 268 (Zif268; Okuno and Miyashita, 1996; Jones et al., 2001) and nuclear factor κB (NF-κB; Freudenthal et al., 1998; Freudenthal and Romano, 2000). For these five transcription factors, strong evidence is now available supporting their essential function in memory processes (Alberini, 2009).

## NF-κB Transcription Factor in the Nervous System

NF-κB was first characterized in lymphocytes but is ubiquitously expressed in different tissues. In mammals, the Rel/NF-κB family of transcription factors consists of five members: p50, a product of the NF-κB1 gene; p52, a product of the NF-κB2 gene; p65 or RelA; c-Rel; and RelB. The Rel/NF-κB family also shares the Rel homology domain containing the following structural regions: DNA binding, dimerization, nuclear localization, and inhibitor κB (IκB) interaction sites. Members of the Rel/NF-κB family bind to DNA, recognizing the consensus decameric sequence 5′-GGGPuNNPyPyCC-3′, designated as the κB site. Different dimers bind with diverse affinities to the distinct κB sites. In addition, gene transcription can be activated or repressed depending on the dimer composition. For example, heterodimers between Rel and NF-κB 1 or NF-κB 2 are activators while homodimers of NF-κB 1 and NF-κB 2 are repressors. Evidence for the existence of Rel/NF-κB members has been found in different species such as humans, rats, mice, chicks, *Xenopus*, *Drosophila*, *Anopheles*, honeybees, crabs, oysters and *Aplysia*, indicating that this transcription factor family is evolutionarily conserved. At variance with most of the transcription factors that are normally found in the nucleus, inactive NF-κB dimers are usually retained in the cytoplasm by binding to IκB inhibitory protein. IκB occludes the nuclear localization signal (NLS) of NF-κB subunits (Hayden and Ghosh, 2008). Upon stimulation, the IκB kinase complex becomes activated and phosphorylates IκB, leading to its ubiquitination and subsequent degradation. In the canonical pathways for NF-κB activation, this degradation allows NF-κB to translocate to the nucleus and regulate the expression of its target genes. NF-κB is expressed in the nervous system both in neurons and non neuronal cells such as glia. The subunits most commonly expressed in neurons are p50-p65 heterodimers and p50 homodimers. NF-κB has been found in pre- and postsynaptic sites (Kaltschmidt et al., 1993; Guerrini et al., 1995; Meberg et al., 1996; Freudenthal and Romano, 2000). The presence of inducible NF-κB in synapses led to the hypothesis that NF-κB is locally activated by synaptic activity and then, in this activated state, is retrogradely transported to the nucleus to regulate gene expression. This fact would confer to this transcription factor a dual function as synaptic activity detector and as transcriptional regulator.

Until now, a number of NF-κB target genes have been found in neurons, and many of them are related to neural plasticity and memory. These include the genes that codify for the following proteins: calcium/calmodulin protein kinase II delta (CaMKIIδ) (see below, Federman et al., 2013), insulin-like growth factor 2 (IGF2; Schmeisser et al., 2012), Zif268 immediate-early gene (Zhou et al., 2003), C/EBP delta (Liu et al., 2007), brain-derived neurotrophic factor (Saha et al., 2006), neural cell adhesion molecule (NCAM; Simpson and Morris, 2000), inducible and neural nitric oxide synthetase (NOS; Morris et al., 2003), cAMP dependent protein kinase A (PKA) α catalytic subunit (Kaltschmidt et al., 2006), protein kinase C (PKC) δ (Suh et al., 2003), and amyloid precursor protein (APP; Song and Lahiri, 1998). NF-κB is widely distributed in the central nervous system (CNS) in regions such as the hippocampus, cerebral cortex, and cerebellum. It is activated upon glutamatergic excitatory synaptic transmission by signals involved in neural plasticity such as depolarization and transient increment of intracellular Ca^2+^ through the Ca^2+^-dependent signaling and the activation of CaMKII (Meffert et al., 2003). NF-κB is also activated in response to dopamine and by neuropetides such as angiotensin II (Frenkel et al., 2002) and by NCAM (Krushel et al., 1999).

## NF-κB in Neural Plasticity and Memory

Long-term potentiation (LTP) and long-term depression (LTD) are models of synaptic plasticity that increase or decrease, respectively, the synaptic efficacy. The induction of *in vivo* LTP in hippocampus increased the expression and the activation of NF-κB (Meberg et al., 1996; Freudenthal et al., 2004). Accordingly, the inhibition of NF-κB reduced LTP and impaired LTD (Albensi and Mattson, 2000). Thus, NF-κB is required in these experimental processes of synaptic plasticity which are considered good models of the synaptic changes occurring during memory storage.

The first evidence of the involvement of NF-κB in memory was obtained in invertebrates, in the context-signal memory of the crab *Chasmagnathus*. In this model, in which contextual cues are associated with a danger stimulus, the activation of NF-κB highly correlates with LTM formation (Freudenthal et al., 1998; Freudenthal and Romano, 2000) while inhibition of this transcription factor impairs memory formation (Merlo et al., 2002). Further studies found NF-κB involved in memory processes in rodents, in different learning tasks such as fear startle potentiation (Yeh et al., 2002), Inhibitory Avoidance (IA; Freudenthal et al., 2005), radial arms maze (Meffert et al., 2003), contextual fear conditioning (FC; Lubin and Sweatt, 2007), conditioned place preference (Yang et al., 2011) and novel object recognition (NOR; Federman et al., 2013).

In the following sections, we will review a series of recent studies in rodents describing the involvement of NF-κB, not only in the memory formation, but also in the different phases of memory, its persistence and its role at the synapse.

## NF-κB in Memory Consolidation

Initially, evidence for the role of Rel/NF-κB in rodents was obtained studying fear-potentiated startle conditioning in rats (Yeh et al., 2002). In this task, trained animals received 10 light stimuli paired with a footshock, and controls received a pseudorandom presentation of the same number of light stimuli and footshocks. In a testing session, the conditioned stimulus was presented together with a white noise and the fear-potentiated startle was evaluated. Using this model, an increment of NF-κB DNA-binding activity was found by electrophoretic mobility shift assay at 2–6 h after training in amygdala but not in hippocampus or cerebellum. IKK activation was also found at 10 and 30 min after training, decreasing to basal levels at 90 min. The intra-amygdala inhibition of NF-κB before training significantly reduced potentiated startle at LTM testing, supporting the idea that NF-κB activation in amygdala is required for memory storage. A further study in this model revealed that FC induced an association between NF-κB and the acetyl transferase CREB binding protein (CBP), resulting in an increase in acetylated p65. The administration of general deacetylase inhibitors prolonged the acetylation state of p65 in amygdala and facilitated LTM (Yeh et al., 2004).

**Figure 1:**
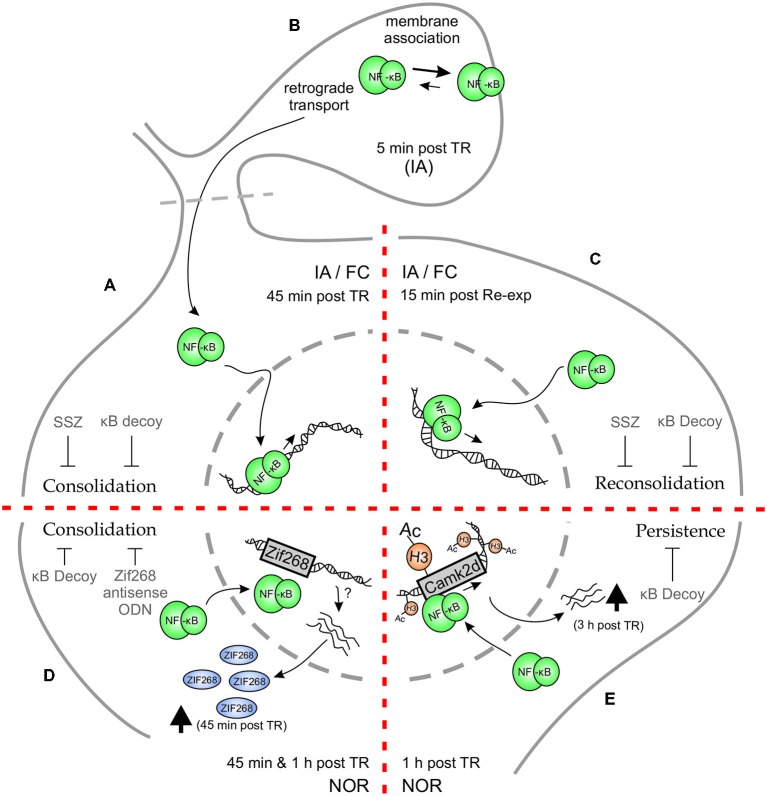
**Schematic representation of Hippocampal NF-κB involvement in different phases of memory for different memory tasks. (A–C)** Inhibitory Avoidance (IA) and Fear Conditioning (FC). **(A)** Both in IA and in FC, training (TR) induces an increase in hippocampal NF-κB activity 45 min after TR. Administration of NF-κB inhibitory drugs—κB decoy or sulfasalazine (SSZ)—in hippocampus disrupts long-term memory (LTM) consolidation. **(B)** When hippocampal synaptosmal preparations were analyzed, a membrane association of NF-κB was observed 5 min post TR in IA paradigm, postulating that synaptic NF-κB not only acts as a retrograde messenger but also has a localized function as well. **(C)** Memory reactivation induces an increase in hippocampal NF-κB activity 15 min after re-exposure to the TR context (Re-exp), both in IA and FC. Hippocampal κB decoy or SSZ administration also impairs LTM reconsolidation in both tasks. **(D–E)** Novel Object Recognition (NOR). **(D)** Training in a NOR paradigm elicits an increment in hippocampal NF-κB activity both at 45 min and 1 h after TR. Moreover, NOR training induces an increment in hippocampal Zif268 protein, which is prevented by κB decoy administration. Both κB decoy and Zif268 antisense oligodeoxinucleotide (ODN) administration in hippocampus impair long-term recognition memory. **(E)** Strong TR elicits a persistent form of NOR memory which involves an increment in histone (H3) acetylation 1 h after TR, that is not observed after weaker trainings. This H3 acetylation is dependent on NF-κB activity, as κB decoy administration prevents it. In particular, CamkIIδ gene was found to be acetylated in its promoter at an NF-κB consensus sequence, which was concomitantly reversed by NF-κB inhibition. CamkIIδ mRNA levels were found to be augmented 3 h post TR. Hippocampal κB decoy administration impaired memory persistence.

The first evidence for the role of Rel/NF-κB in hippocampus was found in an IA task in mice (Figure 1A). The hippocampal formation is a key structure for contextual memories because it is involved in processing and identification of contextual characteristics of different places and in the coding of the unconditioned stimulus associated with a particular place. In this task, animals were placed on an illuminated platform in front of the entrance to a dark compartment. Once the animals entered the dark compartment, they received a mild footshock while controls did not. At LTM testing, trained animals typically showed high latencies to step-through or avoided entering the compartment. Conversely, control animals showed very low latencies to enter. Moreover, we found that the inhibition of this transcription factor by immediate post-training i.c.v. administration of the IKK inhibitor sulfasalazine (SSZ) induced retention deficit in a dose-dependent manner when tested 48 h later (Freudenthal et al., 2005). On the contrary, delayed injections of SSZ at 3 or 24 h post-training did not affect retention. In order to study the effect of direct NF-κB inhibition, we used the κB decoy strategy. The κB decoy consists of a double-stranded DNA oligodeoxinucleotide (ODN) containing a κB consensus sequence that binds to the transcription factor, impeding its action. The κB decoy was administered i.c.v. 2 h pretraining, showing memory impairment. Conversely, injection of the κB decoy with a single base mutation did not affect LTM. Hippocampal NF-κB activity after training increased in shocked animals at 45 min, having returned to basal levels at 2 h post training. These results support the idea that NF-κB activation in the hippocampus is required for the consolidation of contextual features that constitute the conditioned stimulus representation (Freudenthal et al., 2005).

We also studied the dynamics of hippocampal NF-κB activation in the consolidation of contextual fear memory (Figure 1A). In this task, mice associate contextual characteristics of the training chamber with a mild footshock. During testing, the percentage of freezing response is determined. Similar to the results obtained in IA, an activation of hippocampal NF-κB at 45 min was observed after the three-trials training (de la Fuente et al., 2014). The intra-hippocampal administration of SSZ induced amnesic effects, which were revealed by further testing sessions at 24 h and 2 weeks after training (de la Fuente et al., 2014), suggesting a permanent amnesic effect. These findings support that NF-κB is involved in contextual fear memory consolidation in the hippocampus.

IA and FC both entail the presence of an aversive reinforcement, the mild footshock that acts as unconditioned stimulus. At this point, one possible interpretation that could be formulated is that NF-κB is specifically involved in memories associated with aversive and stressful stimuli. NOR is a widely investigated form of declarative memory. It refers to the ability of determining whether a newly found item or object has been previously encountered (Squire et al., 2007). In the classical variant of the task, the animal is exposed during training to two identical objects and is allowed to explore them for a defined time period. During testing, animals are exposed to two different objects, one of them is identical to the training objects and the other is a novel object. The time of exploration of the two objects is then determined to obtain a discrimination index, taking advantage of the rodent’s natural predisposition to explore novel objects more thoroughly. In the NOR, on the one hand, aversive stimuli are absent but on the other hand, the involvement of the hippocampus in this task is a matter of debate (Warburton and Brown, 2010). We then tested whether NF-κB is activated in hippocampus during NOR memory consolidation and found that this transcription factor is active in the nucleus of hippocampal cells at both 45 and 60 min. In other experiment, the infusion of κB decoy in the dorsal hippocampus after training drastically impaired object discrimination when tested 24 h post training (Federman et al., 2013; Zalcman et al., 2015; Figure 1C). All in all, these experiments support both the participation of NF-κB in NOR memory consolidation and the involvement of the hippocampus in this task.

Transcription factors such as NF-κB are involved in the first step of gene expression regulation. These factors are already present at the moment of neuronal activity induced by training. The activation of this kind of transcription factors allows for a second step in gene regulation by induction of immediate-early genes (IEGs) whose protein products are, in turn, transcription factors. Among these IEGs, *zif268* has been found to play a critical role in LTM formation and reprocessing after retrieval. NF-κB binding to the *zif268* promoter region has been identified (Thyss et al., 2005), supporting that NF-κB is involved in its expression. We recently found that Zif268 protein expression was induced 45 min after NOR training in the hippocampus and that the post-training intrahippocampal administration of ODN antisense of the Zif268 mRNA impaired recognition memory. In addition, we found that NF-κB inhibition by κB decoy ODN significantly reduced the training-induced Zif268 increment, indicating that NF-κB is involved in the regulation of Zif268 expression in the hippocampus during recognition memory consolidation (Zalcman et al., 2015; Figure 1C).

## NF-κB in Memory Reconsolidation

In the classical postulation, memory is stored in its definitive long-term form once consolidated few hours after acquisition. During this process, memory becomes resistant to different disruption treatments. However, an important body of evidence has challenged this assumption, supporting the view that memory is more dynamic and can be modified when reactivated by a reminder during retrieval. Under these circumstances, a new period of lability followed by a consolidation-like process takes place. This phenomenon, known in literature as reconsolidation, has been under intense research for the last 15 years (Dudai, 2012).

In an initial report in mammals on the role of NF-κB in memory reconsolidation, we found that NF-κB inhibition in the hippocampus by SSZ or κB decoy after memory reactivation impaired retention of IA memory in mice (Figure 1D). In contrast, mutated κB decoy had no effect. Furthermore, we found NF-κB activation in the hippocampus, with a peak 15 min after memory retrieval (Boccia et al., 2007). This activation was faster than the one previously found in the hippocampus during consolidation of the same task, with a peak at 45 min (Freudenthal et al., 2005), suggesting a temporal signature for both processes. In contextual FC, when animals are re-exposed for 5 min to the training context 1 day after training (once memory consolidation is fully achieved), a labilization-reconsolidation process takes place. During this process, we found that after an initial inhibition, NF-κB showed a peak of activation in mice hippocampus 15 min after the animal was removed from the context (Figure 1D), which is the same time point at which NF-κB was activated after retrieval in the IA task. The infusion of κB decoy immediately after a brief re-exposure caused memory impairment when animals were tested a day after re-exposure, but not 4 h after re-exposure or when mice were not re-exposed. The memory deficit was present even when the mice were tested 2 weeks later, supporting a permanent impairment of memory (de la Fuente et al., 2011). All these findings led us to postulate that NF-κB is activated after memory reactivation as part of the molecular mechanisms involved in memory reconsolidation. Furthermore, the intra-hippocampal administration of the calcineurin phosphatase inhibitor FK506 previous to the re-exposure session induced NF-κB activation and a concomitant memory enhancement that was reverted by the administration of SSZ. Calcineurin actually acts constraining memory in both consolidation and reconsolidation by NF-κB inhibition (de la Fuente et al., 2014).

Lubin and Sweatt (2007) also found evidence on the role of the hippocampal NF-κB pathway in FC reconsolidation in rats, supporting the direct involvement of IKK, the protein kinase that activates NF-κB, in histone phosphorilation in the nucleus. In a recent study, Si et al. (2012) demonstrated that inhibition of NF-κB after memory reactivation in the basolateral amygdala impairs the retention of cued auditory FC. This latter finding supports that NF-κB is not only acting in reconsolidation in the hippocampus but also in amygdala for hippocampal independent tasks. Memory impairment by NF-κB inhibition in reconsolidation was also found in the morphine-induced conditioned place preference task (Yang et al., 2011). Thus, the role of NF-κB in memory reconsolidation is supported in different tasks and different areas of the brain.

## Knock-Out Mice for NF-κB Pathway

The use of gene-targeting strategies in mice gave further support to the role of NF-κB in learning and memory. Mice with a null mutation of p50 showed acquisition deficits in an active avoidance task (Kassed et al., 2002) but, strikingly, normal LTM 24 h after training. Similarly, in another report short-term but not LTM deficits were found in p50 deficient mice (Denis-Donini et al., 2008). Although these results would suggest that p50 is not required for LTM formation, the general effect of the p50 knock-out on κB-dependent transcription is not easy to predict, as this subunit is a component of both a transcriptional activator (p65/p50 heterodimer) and a repressor (p50/p50 homodimer). However, more recent studies of p50 knock-out mice showed that the absence of this NF-κB subunit produced late phase LTP impairment but not early phase LTP impairment, and deficits in long-term spatial memory in the Morris water maze. These results support the hypothesis that the p50 subunit is required in long-term plasticity and spatial memory in the hippocampus (Oikawa et al., 2012).

Baltimore and colleagues have systematically deleted individual NF-κB subunits in transgenic mice. Deletion of the p65 subunit is lethal during embryogenesis (Beg et al., 1995) but the knock-out can be rescued by concurrent deletion of type 1 TNF receptors (Alcamo et al., 2001). The brains of p65/TNFR1 double knock-out mice appear normal under general examination and no obvious behavioral abnormalities were described. With this mutant, Meffert et al. (2003) analyzed memory performance using the radial-arm maze with both the spatial and non-spatial (cued) version. In the spatial version of the task, the external end of all the eight arms had food pellets and spatial clues external to the maze were available. In order to optimize food rewards mice had to remember and to avoid the already visited arms. p65/TNFR1 KO mutants made more errors than p65^+/+^/TNFR1^−/−^ siblings during the first sessions, but in the last sessions no differences in the level of errors were found. In the non-spatial cued version of the task, no external clues were available and only the arms that were illuminated had food pellets. No performance deficit was found in the cued version. The spatial version of this paradigm depends on hippocampus whereas this area is not involved in the non-spatial version. Thus, this result suggests that p65 is required in the hippocampus for neural plasticity necessary in spatial information storage.

Levenson et al. (2004) studied the effect of genetic deletion of c-Rel, a member of the NF-κB family, in FC. In the cued version, animals received a white noise paired with a foot-shock in the training chamber. During testing, freezing response to the noise was evaluated in a different chamber. No differences between c-Rel knock-out and wild type mice were found. However, in the contextual version of the task, in which the training chamber was paired with foot-shock, and the testing was performed in the same chamber, the c-Rel knock-out mice showed impaired LTM. Therefore, c-Rel protein seemed to be necessary for the contextual FC, which is hippocampal dependent, and not required for the cued version in which the hippocampus is not necessary. In a more recent work, the same laboratory found that c-rel^−/−^ mice displayed significant deficits in freezing behavior 24 h after training for contextual FC, but showed normal freezing behavior in cued FC and in short-term contextual FC. Moreover, c-rel^−/−^ mice showed impairment in NOR task. These results indicate that c-rel^−/−^ mice have impaired hippocampus-dependent memory formation (Ahn et al., 2008).

In another study, Kaltschmidt et al. (2006) generated a double transgenic mouse with the expression of tetracycline transactivator (tTA) under the CamKIIa promoter, which confers postnatal expression restricted to the forebrain. The second transgene includes a TetO promoter, regulated by tTA, which allows expression of an IκB super-repressor (non-phosphorylatable form of IκBα). This genetic manipulation caused a general inhibition of NF-κB in forebrain neurons. Treatment with doxycyline rescues the NF-κB deficiency. The performance of this double transgenic mouse without doxycycline (NF-κB deficient) was tested in the hippocampal dependent Morris water maze. The double transgenic animals showed higher latencies during training to reach to the platform than controls, and a deficit in the transfer test without the platform, in which the time spent searching in the correct quadrant was determined. However, no differences were found between these NF-κB-deficient animals and doxycycline-treated animals (NF-κB-rescued), probably due to a nonspecific influence of the drug on memory formation. Besides the behavioral experiments, authors also showed that LTP and LTD were reduced in the Schaffer-collaterals hippocampal path. Notably, they found a down-regulation of the junD gene and the N-CAM gene in these double transgenic mice. In this same study, gene profiling experiments and analysis of regulatory regions identified the α catalytic subunit of PKA as a NF-κB target gene (Kaltschmidt et al., 2006).

In conclusion, most of the studies on genetic manipulation of the NF-κB pathway reviewed here point to an important role of this transcription factor in memory, particularly in hippocampal-dependent tasks.

## Epigenetic Regulation of Persistent Memories

Epigenetic mechanisms regulate the magnitude and the extent of the gene expression pattern induced by learning. The genome of all cells is packaged in the chromatin, a structure comprising DNA and associated proteins, the histones, which determine the different degrees of DNA compaction in its basic structure, the nucleosome. Epigenetic marks consist of modifications of the chromatin structure, which affects transcription of genes. These marks may be post-translational modifications of histones, such as acetylation, phosphorylation, ubiquitination and methylation, as well as changes in the methylation patterns of DNA cytosine residues. Other epigenetic mechanisms include histone variants incorporation to nucleosomes, nucleosome remodeling, and changes in the position of the chromosome in relation to pores in the nuclear envelope (Raisner and Madhani, 2006; Draker and Cheung, 2009; Kundu and Peterson, 2009). All these epigenetic processes occur in an interdependent and coordinated manner, in order to regulate the expression of different parts of the genome (Mehler, 2008).

Previous studies showed that epigenetic mechanisms are involved in memory formation and in particular, in recognition memory. The histone acetyl transferase (HAT) CREB-binding protein (CBP)/p300 is involved in recognition memory consolidation (Oliveira et al., 2011). For instance, the HAT activity domain of CBP is required for NOR memory consolidation (Korzus et al., 2004; Wood et al., 2005) and knock-out mice of the cbp gene show deficits in the NOR task (Alarcón et al., 2004). Furthermore, by the use of specific drugs, it has been found that p300 activity is required for memory formation (Marek et al., 2011; Oliveira et al., 2011; Federman et al., 2013). As was previously mentioned, NF-κB is able to recruit CBP/p300 and to induce histone acetylation in promoter regions containing κB sites (Pradhan et al., 2012).

We hypothesized that NF-κB-dependent histone acetylation during memory consolidation is a molecular feature particularly required for the formation of enduring memories. To test this hypothesis we used three different training protocols: one group of animals received a weak training (3 min of object exploration) which did not induce LTM; another group received a standard training (10 min) that induced LTM which lasts for 24 h but not for 1 week; and the last group received a strong training (15 min) that induced LTM that lasts for 7 days. We found an induction of general histone H3 acetylation of the chromatin in the hippocampus only in animals trained with the strong training protocol. Therefore, these data suggest that the consolidation of more persistent memories differ at the molecular level from the less enduring memories by the induction of this epigenetic mechanism. Next, we explored if the increment in histone acetylation is dependent on NF-κB (Figure 1E). We found that NF-κB inhibition by κB decoy impaired memory persistence and concomitantly, prevented the induction of general H3 acetylation (Federman et al., 2013).

Following up on these findings, we investigated if specific genes involved in memory consolidation showed histone acetylation changes. In particular, we studied the promoters of *zif268* as an instance of immediate-early gene (Davis et al., 2003; Soulé et al., 2008) and calcium/calmodulin kinase II δ (*camk2d*) as an instance of late gene (Sirri et al., 2010; Lucchesi et al., 2011). We found a significant increase in histone H3 acetylation at a specific NF-κB-regulated promoter region of the *camk2d* gene, which was reversed by NF-κB inhibition (Figure 1E). This H3 acetylation increment led to CaMKIIδ mRNA induction 3 h after strong training, but not after weaker training protocols (Federman et al., 2013). No changes in histone acetylation were found in the promoter of the immediate-early gene *zif268*. This work presents a molecular link between NF-κB transcription factor activation, epigenetic mechanisms, and late gene expression in the regulation of memory persistence.

## NF-κB in Neural Synaptic Plasticity

In the last years, important experimental results point to a key role of NF-κB in the determination of synaptogenesis (Boersma et al., 2011; Schmeisser et al., 2012), a basic structural process in the formation of the memory trace. In relation with these findings, the IGF2 gene has been recently identified as a novel IKK/NF-κB target. In IKK/NF-κB signaling-deficient neurons, synapse density is reduced and exogenous IGF2 is able to restore synapse density and promote spine maturation within 24 h. This process depends on IGF2 receptor via MEK/ERK activation. These findings illustrate a fundamental role of IKK/NF-κB–IGF2–IGF2R signaling in synapse formation and maturation in adult mice (Schmeisser et al., 2012). In fact, IGF2 has been previously identified as a memory enhancement factor (Chen et al., 2011).

Several results show that NF-κB is present at the synapses (Kaltschmidt et al., 1993; Guerrini et al., 1995; Meberg et al., 1996; Suzuki et al., 1997; Heckscher et al., 2007) and more recently, the role of the transcription factor during memory consolidation at this neuronal compartment has been explored. There is evidence supporting two major hypotheses regarding the transcription factor at this localization. The first one being that the transcription factor plays a role in the synapse-to-nucleus communication, ultimately regulating gene expression (Wellmann et al., 2001). This synapse to nucleus transport is initiated by an increase in calcium that follows the N-Methyl-D-aspartate (NMDA) receptor activation at the synapse (Wellmann et al., 2001; Meffert et al., 2003). A different hypothesis that could work simultaneously with the previous one, is that NF-κB may modulate synaptic function locally (Heckscher et al., 2007; Salles et al., 2014). On the one hand, it has been reported that the dynein/dynactin motor complex mediates the transport of NF-κB to the nucleus along microtubules and that this transport is dependent on an intact NLS (Mikenberg et al., 2007). It was also observed that NLSs from p50 and p65 are recognized by α importins and that such interaction is necessary for the translocation of the transcription factor to the nucleus (Fagerlund et al., 2005). It would be interesting to study the relation of importins with the dynein/dynactin motor complex in the transport/translocation of NF-κB to nucleus. On the other hand, and supporting the second hypothesis, we found that there are two pools of the transcription factor at this site, one free in the synaptoplasm and another one strongly bound to synaptic membranes (Salles et al., 2015; Figure 1B). The quantities of NF-κB for each pool change after training, decreasing in the synaptosomal content and increasing at the membranes 5 min post-training. This supports the idea that the free NF-κB may be either retrogradely transported to the nucleus or be translocated to synaptic membranes. Furthermore, we found that synaptosomal NF-κB activation occurs 5 min post-training during consolidation (Salles et al., 2015). Therefore synaptosomal activation has a different time course than nuclear activation (Figure 1B). This local activation and membrane dynamics support the idea that NF-κB may be important in the synaptic environment during consolidation. As outlined before, NF-κB may be involved in post-translational modifications of other proteins such as acetylation. There is at least one acetyl-transferase that has been described in dendrites and has been reported to interact with NF-κB, ADP-ribosylation factor domain protein 1 (ARD1; Ohkawa et al., 2008). It has been proposed that this protein is implicated in the microtubule dynamics during spine remodeling, suggesting an involvement of NF-κB in this process. Moreover, in the fruit fly neuromuscular junction, NF-κB homolog Dorsal mediates the membrane insertion of glutamate receptors locally at a post-translational level (Heckscher et al., 2007). All this evidence points towards the idea that parallel to the retrograde transport of NF-κB to the nucleus, the transcription factor may play a key role at the synapses during memory consolidation.

## Concluding Remarks

More than a decade after the initial descriptions of NF-κB’s involvement in memory both in invertebrates (Freudenthal et al., 1998; Freudenthal and Romano, 2000) and vertebrates (Yeh et al., 2002, 2004; Freudenthal et al., 2005), an important body of evidence supports now the participation of NF-κB as a key regulator of gene transcription for long-term storage of information in the nervous system. Furthermore, the presence of NF-κB at the synapses and local activation during memory consolidation may directly influence this process through post-translational modifications of other proteins (Salles et al., 2014). The results reviewed here support that the activity of this family of transcription factors is necessary in the hippocampus for the storage and the persistence of memory in different learning tasks, and both in consolidation and reconsolidation. One of the future directions in the research of the NF-κB role in memory should be the precise characterization of the NF-κB function at the synapse. Another direction for future work is to elucidate the effector genes that are regulated by this transcription factor and the specific role of their protein products. Among the target genes of NF-κB, brain derived neurotrophic factor (bdnf), *zif268* and *camk2d* are candidates for future research efforts in the elucidation of the NF-κB-dependent gene regulation of key proteins that allow memory formation and particularly in the case of the latter gene, in the determination of more persistent memories.

## Conflict of Interest Statement

The authors declare that the research was conducted in the absence of any commercial or financial relationships that could be construed as a potential conflict of interest.
